# Characterization and optimization of ArtinM lectin expression in *Escherichia coli*

**DOI:** 10.1186/1472-6750-12-44

**Published:** 2012-08-02

**Authors:** Maria-Cristina S Pranchevicius, Leandro L Oliveira, José C Rosa, Nilton C Avanci, Andréa C Quiapim, Maria-Cristina Roque-Barreira, Maria-Helena S Goldman

**Affiliations:** 1 Departamento de Biologia, FFCLRP, Av. Bandeirantes 3900, Ribeirão Preto, 14040-901, Brazil; 2 Curso de Medicina, UFT, Av. NS 15 s/n (109 Norte), Palmas, 77010-210, Brazil; 3 Departamento de Biologia Celular e Molecular e Bioagentes Patogênicos, FMRP, Av. Bandeirantes 3900, Ribeirão Preto, 14049-900, Brazil; 4 Departamento de Biologia Geral, UFV, Av. Peter Henry Rolfs s/n, Viçosa, 36570-000, Brazil

## Abstract

**Background:**

ArtinM is a d-mannose-specific lectin from *Artocarpus integrifolia* seeds that induces neutrophil migration and activation, degranulation of mast cells, acceleration of wound healing, induction of interleukin-12 production by macrophages and dendritic cells, and protective T helper 1 immune response against *Leishmania major*, *Leishmania amazonensis* and *Paracoccidioides brasiliensis* infections. Considering the important biological properties of ArtinM and its therapeutic applicability, this study was designed to produce high-level expression of active recombinant ArtinM (rArtinM) in *Escherichia coli* system.

**Results:**

The ArtinM coding region was inserted in pET29a(+) vector and expressed in *E. coli* BL21(DE3)-Codon Plus-RP. The conditions for overexpression of soluble ArtinM were optimized testing different parameters: temperatures (20, 25, 30 or 37°C) and shaking speeds (130, 200 or 220 rpm) during induction, concentrations of the induction agent IPTG (0.01-4 mM) and periods of induction (1-19 h). BL21-CodonPlus(DE3)-RP cells induced under the optimized conditions (incubation at 20°C, at a shaking speed of 130 rpm, induction with 0.4 mM IPTG for 19 h) resulted in the accumulation of large amounts of soluble rArtinM. The culture provided 22.4 mg/L of rArtinM, which activity was determined by its one-step purification through affinity chromatography on immobilized d-mannose and glycoarray analysis. Gel filtration showed that rArtinM is monomeric, contrasting with the tetrameric form of the plant native protein (jArtinM). The analysis of intact rArtinM by mass spectrometry revealed a 16,099.5 Da molecular mass, and the peptide mass fingerprint and esi-cid-ms/ms of amino acid sequences of peptides from a tryptic digest covered 41% of the total ArtinM amino acid sequence. In addition, circular dichroism and fluorescence spectroscopy of rArtinM indicated that its global fold comprises β-sheet structure.

**Conclusions:**

Overall, the optimized process to express rArtinM in *E. coli* provided high amounts of soluble, correctly folded and active recombinant protein, compatible with large scale production of the lectin.

## Background

Lectins are proteins displaying at least one non-catalytic domain, which specifically and reversibly binds to mono or oligosaccharides [1]. Lectins are known as being an extremely useful tool for carbohydrate investigation on cell surfaces, for glycoproteins isolation and characterization, and for lymphocytes polyclonal activation. Numerous lectins have been isolated from many organisms ranging from viruses and bacteria to plants and animals, and they are known to play a key role in a variety of biological processes (reviewed in [2]). Plant lectins have many biomedical applications (reviewed in [3]), including targeted drug delivery (reviewed in [4]) and therapy against several kinds of tumors and infections [5].

ArtinM is a d-mannose-binding lectin from seeds of *Artocarpus integrifolia* that stimulates macrophages and dendritic cells to produce IL-12 [6], an activity triggered by the ArtinM interaction with the N-glycans of TLR2 [7], and is able to induce Th1 biased immune response. As a consequence, ArtinM administration to mice has been shown to confer resistance to Leishmania [6,8], and *Paracoccidioides brasiliensis*[7] infections. The lectin ArtinM is also capable of inducing neutrophil haptotactic migration mediated by the simultaneous interaction of its carbohydrate recognition domains (CRDs) with cell surface N-glycans (linked to the CXCR2 molecule) [9] and extracellular matrix N-glycans (linked to laminin) [10-12]. An amplification loop for the *in vivo* ArtinM inflammatory activity is provided by mast cell degranulation, which is most likely due to the lectin interaction with glycans on FcRI [13]. In addition, ArtinM is able to accelerate the process of wound healing and epithelial tissue regeneration [14]. Therefore, ArtinM has biomedical applications and is a potential pharmaceutical agent. In this study we have aimed to produce high-level expression of active soluble rArtinM in *E. coli* system.

## Results and discussion

### Optimization of soluble rArtinM expression in *E. coli*

The number of recombinant proteins used for therapeutic applications has increased dramatically [15]. In this work, the ArtinM coding region was amplified by PCR, using as template the cDNA clone pLL29 described previously [16]. The primers used were designed to create an *NdeI* and a *BamHI* sites at the initiation and termination codons, respectively. The amplified product was about 460 bp (not shown), which is in accordance with the length of the ArtinM coding region (453 bp). This PCR fragment was digested with *NdeI* and *BamHI*, and cloned into the *NdeI* and *BamHI* sites of the pET29a(+) expression vector. The resulting construction was confirmed by restriction analysis and sequencing (not shown) and named pET29-ArtinM.

**Figure 1:**
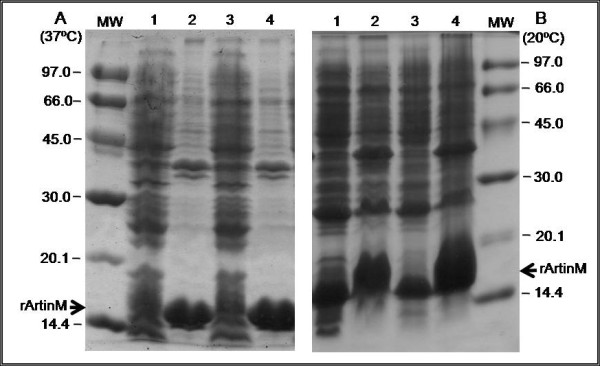
**Optimization of ArtinM expression.** SDS-PAGE analysis of rArtinM expression after **(A)** 1 mM IPTG induction at 37 °C and 220 rpm and **(B)** 0.4 mM IPTG induction at 20 °C and 130 rpm. Lanes 1A and 2A - soluble and insoluble bacterial lysate, respectively, after 4 hours of induction; Lanes 3A and 4A - soluble and insoluble bacterial lysate, after 19 hours of induction. Lane 1B and 2B - insoluble and soluble bacterial lysate, respectively, after 4 hours of induction; Lanes 3B and 4B - insoluble and soluble bacterial lysate, respectively, after 19 hours of induction. Each lane contains 3 μg of total protein. MW molecular weight markers in kDa (LMW – Amersham).

Considering recombinant protein solubility as an indication of its correct folding and activity, our goal was to establish the conditions to obtain high production of soluble protein. Therefore, pET29-ArtinM was introduced in *E. coli* BL21-CodonPlus(DE3)-RP, a strain that contains the T7 expression system and extra copies of the *argU* and *proL* tRNA genes. This strain was chosen because the ArtinM sequence analysis revealed several rare codons (not shown). In our study, different conditions were assayed to determine those able to provide optimal overexpression of soluble ArtinM and four parameters were tested: temperature and shaking speed during induction, concentration of the induction agent (IPTG) and period of induction (for details see Methods). These four parameters were shown to be important in affecting the amount and the solubility of rArtinM. Figure 1 shows the comparison between the results obtained in two different conditions: one in which large amounts of rArtinM was produced (incubation at 37°C, at a shaking speed of 220 rpm, induction with 1.0 mM IPTG for 19 h), but in a insoluble form (Figure 1A), and the optimized conditions (incubation at 20°C, at a shaking speed of 130 rpm, induction with 0.4 mM IPTG for 19 h), in which the highest amount of soluble rArtinM was produced (Figure 1B).

**Figure 2:**
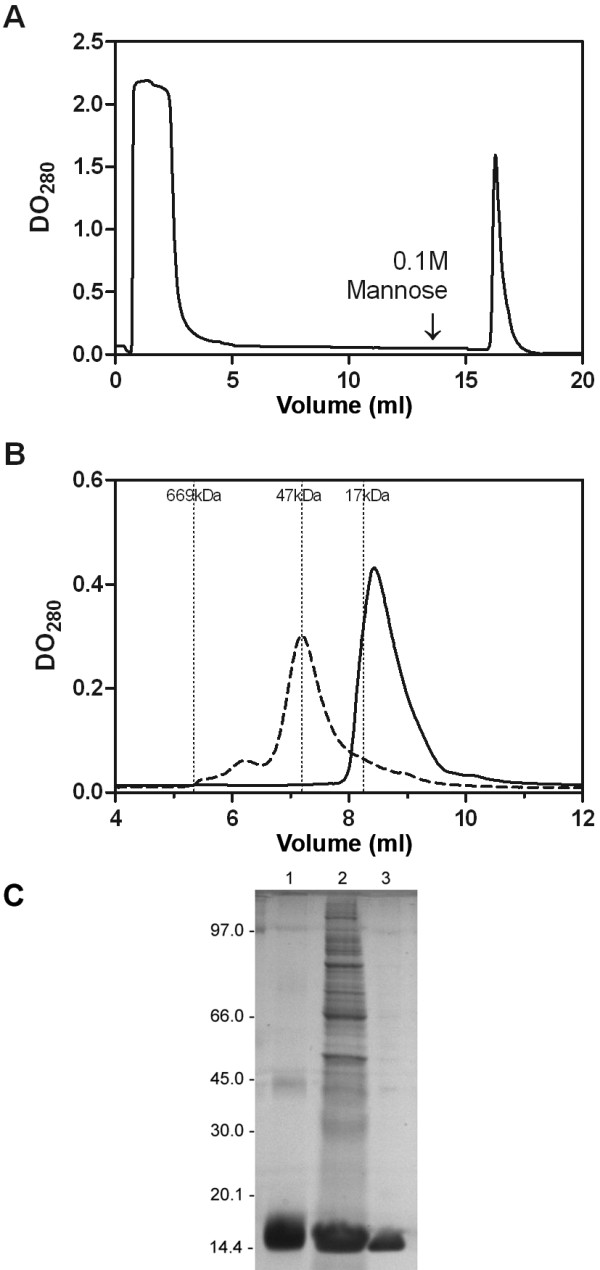
**Purification of rArtinM.A)** Elution profile of rArtinM on d-mannose-Sepharose affinity column. The solid line corresponds to protein elution as monitored by measuring absorption at 280 nm. The arrow shows the d-mannose load. **B)** Size exclusion chromatogram showing jArtinM (dashed line) and rArtinM (solid line) elution. Positions of molecular weight standards are marked. **C)** SDS-PAGE analysis of rArtinM. Lane 1 - purified jArtinM loaded as control. Lane 2 - whole bacterial cell lysate. Lane 3 - rArtinM purified by affinity column. Each lane contains 3 μg of protein.

Taking advantage of the specificity of the carbohydrate recognition property of ArtinM, the recombinant lectin was purified from the *E. coli* lysate by affinity chromatography on a d-mannose column, and was eluted with 0.1 M d-mannose in PBS, providing the profile showed in Figure 2A. Such purification process by itself certifies that the sugar binding activity of rArtinM was preserved. Measurements by BCA assay (see Methods) revealed an average yield of 22.4 mg rArtinM per liter of culture. The rArtinM has been analyzed through TSK-G2000sw gel-filtration column and the elution profile showed a single peak of 16 kDa, consistent with the monomer molecular mass. Meanwhile, as expected, the jArtinM was eluted in a volume compatible with the tetramer molecular mass (Figure 2B). The homogeneity of rArtinM was confirmed by SDS-PAGE (Figure 2C). Taken together, these results show that the rArtinM produced in *E. coli* is monomeric and capable to bind d-mannose.

Production of recombinant proteins in active and highly purified form for biomedical research, biotechnology, and pharmaceutical industry is a huge challenge. The major and frequent difficulty in expressing a heterologous protein in a bacterial system concerns the tendency of the recombinant protein to become insoluble [17]. Our data shows that in the optimized conditions here defined (incubation at 20°C, at a shaking speed of 130 rpm, induction with 0.4 mM IPTG for 19 h), soluble and active ArtinM is produced in large quantities in the *E. coli* expression system.

### rArtinM and jArtinM have similar primary structure

**Figure 3:**
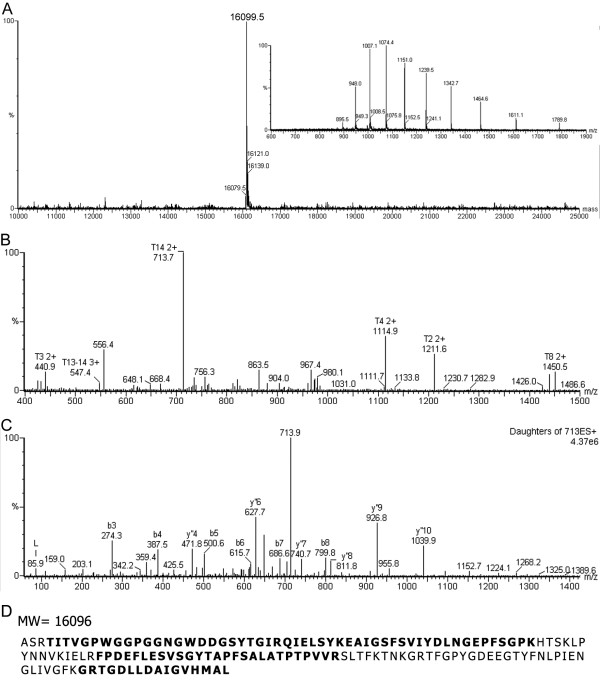
**Mass spectrometric analysis of rArtinM.A)** Molecular mass of rArtinM (of 16099.5 Da) was determined by ESI-triple quadrupole mass spectrometer. The insertion in figure shows protonated envelop of ions which were de-convoluted to Mw using MaxEnt 1 algorithm. **B)** Mass fingerprint obtained from tryptic digested rArtinM in solution was analyzed by ESI-triple quadrupole scanning from 400 to 1500 amu in the positive ion mode for detection of protonated peptides. **C)** Each tryptic peptide was subjected to collision-induced dissociation to produce a fragment ion pattern and the amino acid sequence was deduced from fragment ions type *b* and *y*, as an example, spectrum of tryptic peptide m/z 713.7 [M + 2 H^+^] which corresponds to the C-terminal peptide. Other tryptic peptides are listed in Table 1. **D)** Amino acid sequence of rArtinM determined by Edman degradation (N-terminal, residues 1–20) and tryptic peptides by mass spectrometry (bold).

rArtinM was characterized by mass spectrometry and N-terminal amino acid sequencing by automated Edman degradation. Electrospray ionization mass spectrometry (ESI-MS) has been regularly used to characterize recombinant proteins, since it is a rapid and precise method for determining molecular mass of proteins and peptides and can be used to validate protein sequences [18,19]. Therefore, a sample of purified rArtinM has been analyzed by ESI-triple quadrupole – mass spectrometer. MaxEnt 1 algorithm was used for de-convolution of multiple envelop ions which determined that rArtinM has a molecular mass of 16,099.5 Da (Figure 3A). It is in good agreement with the molecular mass determined for jArtinM (masses of 16,101.5 and 16,114.5 Da for the two major isoforms - data not shown) and the average molecular mass (16,124.11 Da) calculated from the amino acid sequence [20]. Amino acid sequencing of peptides derived from trypsin digestion of rArtinM, performed by ESI-MS peptide mass fingerprinting (PMF) and collision induced dissociation (CID-MS/MS) has covered 41% of the total ArtinM amino acid sequence (Figure 3B, Table 1). The spectrum of C-terminal peptide (Figure 3C) confirmed that rArtinM C-terminal sequence was equal to jArtinM (Table 1). N-terminal sequencing showed that the first 20 amino acids are in accordance with the sequence of jArtinM, except for the substitution of glutamine (Q) for arginine (R) at residue 3 (Figure 3D), which was confirmed by Edman degradation and mass spectrometry. This substitution was due to an unintentional mutation introduced at the cloned sequence, as verified by DNA sequencing. Mass spectrometry detected this new trypsin cleavage site correspondent to a tryptic peptide at residues 4 to 27 (Table 1). N-terminal sequencing by Edman degradation of rArtinM indicates that the recombinant protein was not acetylated at the N-terminal, a modification that was found in jArtinM [20]. The absence of N-acetylation (−42 Da) at the N-terminal and replacement of Q for R (+28 Da) account for a molecular mass of 16,113.5, which is close to native isoforms (16,101.5 and 16,114.5 Da).

**Table 1:** Tryptic peptide mass mapping obtained from rArtinM

***m/z***	***M***_**r**_	**Residues**	**Sequence**
440.9 [M + 2 H^+^]	879.47	29-35	(R)QIELSYK(E)
547.4 [M + 3 H^+^]	1,637.86	135-150	(K)GRTGDLLDAIGVHMAL(−)
713.7 [M + 2 H^+^]	1,424.73	137-150	(R)TGDLLDAIGVHMAL(−)
1,114.9 [M + 2 H^+^]	2,226.08	36-56	(K)EAIGSFSVIYDLNGEPFSGPK(H)
1,211.6 [M + 2 H^+^]	2,419.12	4-27	(R)TITVGPWGGPGGNGWDDGSYTGIR(Q)
1,450.5 [M + 2 H^+^]	2,898.45	72-98	(R)FPDEFLESVSGYTAPFSALATPTPVVR(S)

The absence of the initial methionine at the rArtinM N-terminus, as in the mature jArtinM, was an interesting and unexpected finding. Recombinant proteins produced in *E. coli* cytosol often possess the methionine, corresponding to the translational initiation codon (ATG), at the N-terminus [21]. In a significant fraction of the *E. coli* endogenous cytosolic proteins, this N-terminal methionine residue is excised by a methionylaminopeptidase (MAP) [22]. Biochemical and genetic studies indicated that the major determinant for cleavage by MAP is the amino acid occupying the N-terminal penultimate position or, in other words, the second amino acid [23,24]. According to the generally accepted rules for MAP, one of the highest cleavage probabilities is found when Ala is the second amino acid [24,25], as in the ArtinM sequence. Therefore, it is reasonable to propose that the N-terminal methionine of the rArtinM was efficiently processed in *E. coli* BL21-CodonPlus(DE3)-RP by endogenous MAPs.

### The rArtinM has secondary and tertiary structures equivalent to the jArtinM

Circular dichroism spectra (CD) and fluorescence spectroscopy of rArtinM and jArtinM were obtained in order to evaluate the correct folding of the recombinant protein and determine some structural details (data not shown). The analysis of secondary structure content showed that rArtinM contained predominantly β-sheet structure, as characterized by the positive ellipicity at wavelength 195 nm and the negative ellipticity at 218 nm. Fluorescence measurements were performed in order to verify the presence of tertiary structure, all emission spectra were recorded from 300 – 450 nm with excitation at 280 nm (data not shown). Thus, our CD spectrum and the fluorescence analysis indicated that rArtinM is correctly folded and has a defined conformational structure suitable for comparative functional studies.

### Functional analysis of rArtinM using glycan array

The characterization of the specificities of glycan-binding proteins is of primary importance for a recombinant lectin, and the glycan array has been an important tool for this investigation. The specificity of the fluorescence-labelled lectins was evaluated by binding to the 406 oligosaccharides present on the glycan array available at the Consortium for Functional Glycomics. The glycan array profile for both native (jArtinM) and recombinant ArtinM reveals that both lectin forms recognized with high affinity the same subsets of complex-type biantennary N-glycans containing Manα1-3(Manα1-6)Manβ1-4GlcNAcβ1-4GlcNAcβ (data not shown). This result is coherent with our recent observation that native and recombinant ArtinM interact with equivalent kinetic rates and affinity equilibrium constants to horseradish peroxidase glycoprotein [26], a N-glycosylated protein that contains the trimannoside Manα1-3[Manα1-6]Man, which is a known ligand for jArtinM [27].

### IL-12 inducing property of jArtinM is preserved in rArtinM

**Figure 4:**
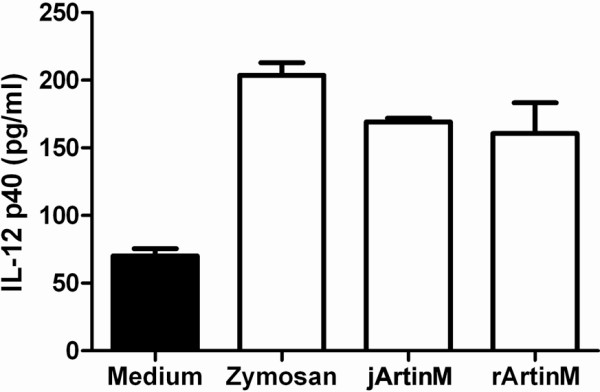
**IL-12 production induced by rArtinM.** Peritoneal macrophages were cultured for 48 hours in the presence or absence of zymosan (10 μg/ml), jArtinM (5 μg/ml), rArtinM (5 μg/ml). The amount of IL-12p40 in the supernatant was determined by capture ELISA.

Functionally, we had previously demonstrated that jArtinM induces IL-12 production by macrophage cell lines, as well as peritoneal and spleen macrophages [6,7]. To determine whether this property of jArtinM was preserved in rArtinM, we next verified the *in vitro* IL-12p70 production by spleen macrophages stimulated with 5 μg/ml of rArtinM (Figure 4). We observed that the rArtinM-stimulated macrophages released IL-12 in culture supernatants in concentrations that were similar to the induced by the native protein, demonstrating that the rArtinM produced in *E. coli* preserved this biological activity exerted by the plant jArtinM. This fact is conceptually acceptable, because IL-12 production is triggered by ArtinM interaction with type 2 toll-like receptor, whose usual agonists are low molecular mass microbial components, unable to enclose more than one site of interaction with the receptor. However, it is expected that ArtinM activities that depend on receptor cross-linking to be triggered, such as mast cell degranulation, will not reproduced by the monomeric recombinant lectin.

## Conclusion

Considering the potential use of ArtinM as an immunotherapeutic molecule, this study was designed to produce high-level of soluble/active rArtinM in *E. coli* system, for both research and pharmaceutical purposes. Here we report a high-yield production of rArtinM lectin using pET29a(+) and BL21-CodonPlus(DE3)-RP as expression system, and its characterization by SDS–PAGE, one-step purification through immobilized d-mannose affinity chromatography, circular dichroism (CD), fluorescence spectroscopy, glycoarray analysis and IL-12 production. Several evidences indicate that the final product, rArtinM, is correctly folded, biochemically active and endowed of biological properties exerted by the plant lectin ArtinM. Taken together, our data provides evidences that rArtinM will be a useful tool for future biomedical studies and that *E. coli* expression system is appropriate to produce large quantities of functional ArtinM for industrial purposes.

## Methods

### Cloning the ArtinM coding region in a *E. coli* expression vector

The coding region of the ArtinM lectin was amplified by PCR using as template the cDNA clone pLL29 previously described [16]. The primers (forward -5’gaaggtgaatcatATGgcgagccag3’ and reverse - 5’ggacatattggatccCTAaagtgcc3’) used for cloning the ArtinM coding region introduced the restriction site *Nde*I (underlined) at the initiation codon (capital letters) and the *Bam*HI site (underlined) just after the stop codon (capital letters). These primers were used for amplification with the TripleMaster polymerase (Eppendorf, Hamburg, Germany), under the following PCR conditions: 3 min at 94°C followed by 35 cycles of 1 min at 94°C, 45 s at 55°C, and 1 min at 72°C; the final extension was for 7 min at 72°C. The PCR product was digested with the *Nde*I and *Bam*HI restriction enzymes, separated on a 1% agarose gel and extracted from the gel using a phenol/chloroform protocol. The pET-29a(+) vector was digested with the same two enzymes and purified from a 1% agarose gel. The digested PCR fragment of ArtinM (453 bp) was ligated into the linearized vector pET-29a(+). The resulting vector, named pET29-ArtinM, was confirmed by restriction analyses and sequencing and introduced in *E. coli* BL21-CodonPlus(DE3)-RP cells by electroporation.

### Optimization of ArtinM expression

*E. coli* BL21-CodonPlus(DE3)-RP transformants were inoculated in 5 mL of Luria–Bertani (LB) medium supplemented with kanamycin 50 μg/mL and cultivated overnight at 37°C. This pre-culture was used to inoculate (1:100) a 100 mL LB medium containing kanamycin, which was cultivated at 37°C until reaching a DO_600nm_ of 0.5-0.6. Then, an aliquot of 10 mL was collected (T0) and IPTG was added. To optimize ArtinM expression, IPTG was tested at different final concentrations (0.01, 0.05, 0.1, 0.2, 0.4, 0.8, 1.0, 1.5, 2.0, 2.5, 3.0, 3.5, 4.0 mM). After the addition of IPTG, each culture was incubated at different temperatures (20, 25, 30 or 37°C), under different shaking speeds (130, 200 or 220 rpm), for different incubation periods (1, 2, 4, 8, 16 and 19 hours). Aliquots (10 mL) were collected at these specific time points to evaluated rArtinM expression. Cells were pelleted by centrifugation at 5,000 × g at 4°C for 5 min, the culture medium was discarded and cells ressuspended in lysis buffer (10 mM Tris–HCl, 300 mM NaCl, 1 mM EDTA, 5 mM DTT, 100 μg/mL lysozyme, 0.5% (v/v) glycerol and protease inhibitors). The homogenates were sonicated 4 times for 30 seconds, with 30 seconds interval between each sonication and then, centrifuged to separate supernatant and pellet. These samples were quantified and analyzed by SDS-PAGE.

For the additional analyses of rArtinM, a pre-culture aliquot of 1.5 mL was used to inoculate 150 mL LB medium. Cells were cultivated at 37°C to an OD_600nm_ of about 0.6. Then temperature was decreased to 20°C and the expression of ArtinM was induced by the addition of 0.4 mM IPTG (Appli-Chem GmbH, Darmstadt, Germany) and cells were allowed to grow for another 19 h at 130 rpm. Cells were harvested by centrifugation at 8,000 × g at 4°C for 20 min. The cell pellets were used immediately for protein purification or frozen in liquid nitrogen and stored at −70°C.

### ArtinM Affinity Purification

jArtinM, extracted from *Artocarpus integrifolia* seeds, was purified as previously described [28]. *E. coli* BL21-CodonPlus(DE3)-RP was used to express rArtinM as described above and soluble proteins were obtained through bacterial sonication and centrifugation at 25,000 × g for 15 min. The supernatants were submitted to affinity-chromatography on a d-mannose column, previously equilibrated at 4°C with PBS containing 0.5 M NaCl. After washing with equilibrating buffer, the adsorbed material was eluted with 0.1 M d-mannose in equilibrating buffer. The obtained preparation was ultradiafiltered against PBS, using YM10 membrane (Amicon Division, W.R. Grace, Beverly, MA). ArtinM preparations contained less than 0.05 ng/ml of bacterial endotoxin, as determined by the Limulus amoebocyte lysate assay (Sigma Chemical Co., St. Louis, MO).

### Protein analyses

Protein quantification was performed through the bicinchoninic acid (BCA) assay (Sigma Chemical Co., St. Louis, MO), using BSA as standard [29]. Protein electrophoresis was carried out by conventional SDS–PAGE and the gels have been stained with Coomassie blue R-250 (Sigma Chemical Co., St. Louis, MO).

### Analytical size exclusion chromatography

Analytical size exclusion chromatography was performed on a TSK-G2000sw (30 cm × 7.8 mm, Tosoh) equilibrated in 50 mM KH_2_PO_4_, 300 mM NaCl, pH 7.4. The column was calibrated with bovine thyroglobulin (669 kDa), immunoglobulin G (157 kDa), ovalbumin (45 kDa), and myoglobin (17 kDa) (Sigma).

### Mass spectrometric analysis of ArtinM

Native and recombinant Artin M were desalted in POROS R2 (Perseptive Biosystem, Foster City, CA) and about 2.5 μg of each sample was directly infused by syringe pump (Harvard Apparatus, Holliston, MA) into a triple-quadrupole mass spectrometer (Quattro II, Micromass, Manchester, UK) equipped with an electrospray ion source. Fifteen scans were collected between 400 and 2000 amu, and the molecular weight was determined after de-convolution of multi-charged ions spectrum by MaxEnt1 algorithm (MaxLynx software v3.3, Micromass, Manchester, UK).

### Peptide mass fingerprint of rArtinM

An aliquot of 2.5 μg of rArtin M was heated denatured and subjected to enzymatic digestion with 0.5 μg of modified trypsin (Promega, Madison, WI, USA) for 24 h at 37°C. The enzyme reaction was stopped with 5 μL of neat formic acid. The tryptic peptides were desalted in POROS R2 (Perseptive Biosystem) previously activated with methanol, equilibrated in 0.2% formic acid and the peptides were eluted in 60% methanol, 5% formic acid. The MS analysis of tryptic peptides was carried out by ESI-triple quadrupole MS (Quattro II, Micromass, Manchester, UK) at the mass range of 400–1500 u.m.a. and the peptide ions were selected to collision induced dissociation (CID-MS/MS) to produce fragments pattern mainly type *b* and *y* which were used for deduction of amino acid sequences.

### Spectroscopic characterization of recombinant ArtinM

Far UV circular dichroism spectra were performed using a Jasco J-810 spectropolarimeter in the wavelength range of 190–280 nm. Measurements were made on the purified ArtinM lectin (native and recombinant) at a concentration of 0.5 mg/mL, using quartz cuvettes of 0.l mm path length. Spectra were typically recorded as the average of 6 scans. CD spectra were obtained in milli-degrees and converted to molar ellipticity. Intrinsic tryptophan fluorescence emission (IFTE) spectra were measured using a SLM-AMINCO 8100c (Spectronic Instruments) between 300 and 450 nm using an excitation wavelength of 280 nm at protein concentration of 5 mg/ml. The excitation and emission slit widths fixed at 4 nm and the photomultiplier tube voltage was 600 V. In all spectroscopic measurements the buffer was saline (150 mM NaCl).

### Glycoarray analysis

A high-throughput screening for identify lectin-ligand interactions was performed by the standard procedure of Core H of the Consortium for Functional Glycomics [30]. Briefly, synthetic glycans functionalized with a spacer and terminating NH2 groups were spotted onto NHS-activated microscope slides (Slide H). Lectins at a concentration of 20–200 μg/mL in a buffer of PBS containing 0.005%–0.5% Tween-20 were incubated on the arrays for 30–60 min. The lectins were tagged with a Fluorescein isothiocyanate (FITC, Molecular Probes). The arrays were washed and immediately scanned for fluorescence using a microarray scanner. Image analysis software was used to quantify the fluorescence intensities at each glycan spot. The data from six replicate spots were averaged to achieve a final value.

### Spleen cell cultures

Suspensions of spleen cells from the C57BL/6 were washed in RPMI-I (RPMI 1640 - Flow Laboratories, Inc., McLean, VA) and treated with lyses buffer (9 parts of 0.16 M ammonium chloride and 1 part of 0.17 M Tris–HCl, pH 7.5) for 4 min. The erythrocyte-free cells were then washed three times in RPMI-I, resuspended in RPMI-C (containing 2 mM L-glutamine, 50 μM 2-mercaptoethanol, 100 U/ml penicillin, 100 μg/ml streptomycin [Sigma-Aldrich] and 5% heat-inactivated fetal calf serum [Hyclone, Logan, UT]), and dispensed in 24-well cell culture plates (1 × 10^7^ cells/well). After 2–4 h incubation at 37°C, the non-adherent cells were removed by exhaustive washing with RPMI-I, and the adherent cells were incubated with jArtinM (5 μg/ml), rArtinM (5 μg/ml), and zymosan (10 μg/ml) – which had been previously boiled for 30 min and washed twice with PBS. After 48 h incubation, the supernatants were harvested by centrifugation and stored at −20°C until IL-12p40 ELISA was performed.

### IL-12p40 ELISA

The levels of IL-12p40 in the macrophage supernatants were measured by capture enzyme-linked immunosorbent assay (ELISA) with antibody pairs purchased from Pharmingen (Pharmingen, San Diego, USA). The ELISA procedure was performed according to the manufacturer’s protocol. The IL-12p40 concentration was determined with reference to a standard curve for murine recombinant IL-12.

## Misc

Maria-Cristina S Pranchevicius and Leandro L Oliveira contributed equally to this work.

## Competing interests

The authors did not declare any competing interests.

## Authors' contributions

MCSP and LLO participated in the design of the study, performed the experiments, analyzed the data and drafted the manuscript. JCR performed the experiments, analyzed the data and drafted the manuscript. NCA analyzed the data. ACP performed the experiments. MCRB and MHSG conceived the study, participated in its design and helped to draft the manuscript. All authors read and approved the final manuscript.
